# Complaint handling in healthcare: expectation gaps between physicians and the public; results of a survey study

**DOI:** 10.1186/s13104-015-1479-z

**Published:** 2015-10-01

**Authors:** R. D. Friele, P. M. Reitsma, J. D. de Jong

**Affiliations:** NIVEL, PO box 1568, 3500 BN Utrecht, The Netherlands; Tilburg University, Tilburg, The Netherlands

**Keywords:** Patient complaints, Complaints handling, Medical errors, Patient satisfaction, Medicine, Communication, Medical claims

## Abstract

**Background:**

Patients who submit complaints about the healthcare they have received are often dissatisfied with the response to their complaints. This is usually attributed to the failure of physicians to respond adequately to what complainants want, e.g. an apology or an explanation. However, expectations of complaint handling among the public may colour how they evaluate the way their own complaint is handled. This descriptive study assesses expectations of complaint handling in healthcare among the public and physicians. Negative public expectations and the gap between these expectations and those of physicians may explain patients’ dissatisfaction with complaints procedures.

**Methods:**

We held two surveys; one among physicians, using a panel of 3366 physicians (response rate 57 %, containing all kinds of physicians like GP’s, medical specialist and physicians working in a nursing home) and one among the public, using the Dutch Healthcare Consumer Panel (n = 1422, response rate 68 %). We asked both panels identical questions about their expectations of how complaints are handled in healthcare. Differences in expectation scores between the public and the physicians were tested using non-parametric tests.

**Results:**

The public have negative expectations about how complaints are handled. Physician’s expectations are far more positive, demonstrating large expectation gaps between physicians and the public.

**Conclusions:**

The large expectation gap between the public and physicians means that when they meet because of complaint, they are likely to start off with opposite expectations of the situation. This is no favourable condition for a positive outcome of a complaints procedure. The negative public preconceptions about the way their complaint will be handled will prove hard to change during the process of complaints handling. People tend to see what they thought would happen, almost inevitably leading to a negative judgement about how their complaint was handled.

**Electronic supplementary material:**

The online version of this article (doi:10.1186/s13104-015-1479-z) contains supplementary material, which is available to authorized users.

## Background

Patients that file a complaint—a statement that one is unhappy or unsatisfied with something [1]—want a genuine apology from their physician, an explanation of what happened and information about proposed changes to prevent recurrences [2–6]. However, patients that file a complaint are often dissatisfied with the response to their complaints [2, 7–9]. Finding ways to handle complaints that are meaningful for patients is more than just the decent thing to do [10–12]. Improving how complaints are handled may help reduce the numbers of financial claims and prolonged legal disputes between patients and their physicians, as well as serving as a source of information for quality improvement [13, 14] and improving patient safety [11, 15, 16].

Several studies attribute the negative experiences of patients with complaints handling to failure by the physicians involved to meet complainants’ expectations [2, 3, 17–20]. Research to date has primarily focused on patients who have submitted a complaint, looking back at their experiences. Another approach, the one taken here, is to look at the expectations of the public in general. Negative expectations may lead to confirmation bias simply because they make it difficult to see things in any other way than expected, as discussed by Oliver [21]. In addition, the expectations of physicians about how complaints are dealt with may play a role. Research in a variety of service contexts, including healthcare, suggests that there are gaps between the expectations of the professionals providing the service and the expectations of the customers receiving the service [12, 22–28]. Discrepancies between the expectations of the public and physicians may lead to mutual misunderstanding and so frustrate communication between the two parties [28]. Understanding the expectations of both physicians and the public and the potential gaps between the two may help to understand why patients’ evaluations of complaints handling are so often negative.

This study aims to answer two questions:

- What are the expectations of complaints handling among the public and physicians?
- Are there differences between the expectations of complaints handling among the public and among physicians?

## Methods

We conducted parallel cross-sectional surveys among physicians and the public and compared the answers of both populations (the questionnaire is available as Additional file 1). This study was performed in the Netherlands, where serious attempts have been made to handle complaints in a way that takes account of the needs of patients. Addressing the complaint with the physician involved is seen as the most preferred way of dealing with a complaint, and the most frequently used way of dealing with a complaint, according to patients’ reports [29, 30]. More formal alternatives are to submit a complaint to complaints committee, which every care provider is obliged to have. These committees were established to provide a patient-oriented complaints procedure. Complaints may also be submitted to a disciplinary board [31] or the health care inspectorate. All of these types of complaints are implied in the questionnaires.

### Data collection

#### Physicians

An Internet questionnaire was sent to 3366 physicians of the panel of physicians maintained by the Royal Dutch Medical Association (KNMG), the professional organization for physicians in the Netherlands. The panel is representative for the population of physicians in the Netherlands and the members of the KNMG. The response rate was 57 % (n = 1935).

#### The public

Another questionnaire was sent to 1422 members of the Dutch Healthcare Consumer Panel [32], a representative sample for the Dutch population in terms of gender and age (≥18). A mixed-method approach was chosen for this panel to avoid selection bias caused by sending the questionnaire only through the Internet. Based upon individual preferences, respondents either received the questionnaire by post or over the Internet. The response rate was 69 % (n = 987). Selective response occurred, especially among those under 30 years of age. Table 1 summarizes respondent characteristics: age, gender and medical discipline for physicians, and age, gender and educational level for the public.

**Table 1:** Sample respondent characteristics

	Public (n = 987) %	Physicians (n = 1935) %
*Gender*		
Female	55	45
*Age*		
<30	1	10
30–45	15	31
46–60	34	43
>60	50	16
*Education*		–
Low (lower vocational training or less)	21	
Medium	49	
High (higher vocational training and university)	31	
*Experience with complaints*		
Did you ever present a complaint about the care provided? (formal or informal)	14.2	–
Did you ever receive a complaint from a patient about the care you provided?	–	78.1

### Questionnaires

**Fig. 1:**
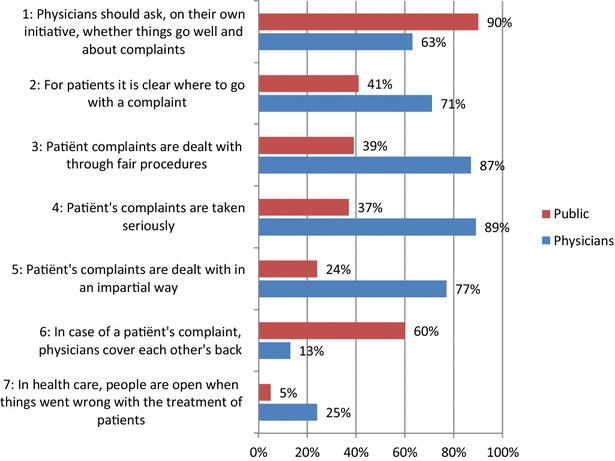
Expectations of complaints handling: percentage of the public and physicians who agree or agree or totally agree

The questionnaires contained seven items on complaint handling, based on earlier qualitative and quantitative research among patients with complaints [4, 33]. Figure 1 lists these items. One item referred to the normative issue of openness among physicians (item 1). The other items referred to the expectations of actual behaviour of physicians and how complaints are handled, covering accessibility (item 2), the procedure (items 3–5) and openness (items 6 and 7). These items were presented to both physicians and the public in the same wording, using a 5-point Likert scale for the response: (1) totally agree, (2) agree, (3) agree nor disagree, (4) disagree, (5) totally disagree. Respondents could also indicate that they had no opinion on the subject; this was treated as a missing value. Missing values ranged from 0.7 % (item 1) to 8.5 % (item 5).

### Statistics

Due to selective non-response, the respondents did not match the reference population. The data from the physicians was therefore weighted for medical discipline to match the distribution of the different types of doctors. The response from members of the public aged below 30 was virtually absent, this age category was combined with the next category, ages 30–45. After this, the data for the public was weighted for age and gender. To be able to assess the potential influence of age on the expectation variables, we tested the relationship between age and expectation using Spearman’s correlation test. Differences between the expectations of physicians and the public were tested using the Mann–Whitney rank-sum tests. The results section will focus on significant differences. Only differences with that are statistically significant (p < 0.01) will be presented without explicitly mentioning the value. The data was analysed using STATA 12.

### Ethics statement

Our study complied with the Helsinki Declaration where applicable. According to the Dutch ‘Medical Research involving human subjects Act’, neither obtaining informed consent nor formal ethical approval for this study was required. No medical interventions were involved and the impact of the questionnaires on daily life was considered minor and thus the welfare and rights of the panel members were protected. Panel members were free to answer the questions or not. The study was reviewed by the research-review board of NIVEL.

## Results

### Experience with complaints

Fourteen percent of the public reported that they ever presented a complaint, whereas 78 % of physicians reported that they at least once received a complaint (Table 1).

Among the public gender nor age were related to having presented a complaint. Educational level was related to having filed a complaint (p = 0.048), the higher the education the more often a complaint was filed. Female physicians less often reported they received a complaint (72 versus 83 % for males), younger physicians also reported less often reported they received a complaint (those under 30: 48 %, between 30 and 45: 78 %, between 46 and 60: 83 % and over 60 85 %). Gender and age are correlated: on average female physicians are younger than male. The effect of age remained significant within females and males, whereas the effect of gender lost significance within three out of four age-categories. Only in the age category 30–45 more males than females reported that they had received a complaint. The medical discipline was not related to the number of physicians reporting to have received a complaint.

### Expectations of complaints handling

The majority of the public (90 %) and physicians (63 %) agree that physicians should take the initiative and ask whether things are going well and inquire about any complaints (Fig. 1). This expectation gap of 27 % points is significant: the public expect more of physicians on the issue of taking the initiative than physicians expect of themselves and their colleagues.

Regarding accessibility, 41 % of the public believe that it is clear where patients should go with a complaint, with older people agreeing more often (age 18–39: 39 % agreed; 40–59: 41 % agreed; >60: 48 % agreed), whereas almost three quarters (71 %) of physicians believe so: a significant expectation gap of 30 % points.

Larger expectation gaps concern the fairness of the complaints procedure: 39 % of the public say that complaints are dealt with through fair procedures and 37 % say that complaints are taken seriously, whereas more than four out of five physicians say so (87 and 89 % respectively). This means that the expectation gaps are 48 and 52 % points respectively. Also, only about one in four (24 %) of the public believe that complaints are dealt with impartially. In contrast, three quarters (77 %) of the physicians think so: a 53 % points expectation gap.

Differences and similarities in the expectations of the public and physicians were found on issues relating to being open when a complaint is filed. More than half (60 %) the public believe that physicians close ranks in the event of a patient complaining; only 13 % of physicians think so. This is a large and serious gap, because it suggests that a little over half the public do not believe in the impartiality of other physicians who might be involved in the complaints procedure.

Finally, a minority of the public (5 %) and physicians (24 %) believe that people are open when things go wrong with the treatment of patients. Older people among the public agreed more often (age 18–39: 1 % agreed; 40–59: 8 % agreed; >60: 9 % agreed). Although this expectation gap is small and significant, the majority of both populations agree that people are *not* open when things go wrong with patient treatment.

When comparing the answers of the public between those that filed and those that did not file a complaint, only few significant differences were found (not in table). Those that had filed a complaint less often (p = 0.025) agreed that complaints are dealt with through fair procedures, they also less often agreed that complaints are taken seriously (p = 0.048). Having received a complaint as a physician is related to their view on complaints handling in five out of the seven items. Physicians that received a complaint more often agreed that for patients it is clear where to go with a complaint. They also more often agreed that the handling of complaints was through fair procedures, that complaints are taken seriously and that complaints are dealt with in an impartial way. They less often agreed that physicians close ranks in case of a patient’s complaint.

## Discussion

### Openness when things go wrong

The majority of both physicians and the public in this study agree that physicians should take the initiative and ask if things are going well and if there are any complaints. The majority of both parties also agrees that openness is not yet common practice. These findings may not come as a surprise. Despite the fact that a movement in the direction of openness is “under way”, as demonstrated by a shared normative view on openness among the majority of physicians and the public, the tendency among physicians has been to cover up and not disclose [11, 14, 34], partially fuelled by fear of litigation [10, 35]. In the Netherlands, a clear code of conduct endorsed by all parties such as the professional organizations, hospitals and insurance companies was only recently issued [36]. This code provides a set of guidelines that advocate openness, swift response, solving possible problems, offering an explanation, admitting mistakes, learning from complaints and giving information about complaints procedures. It will take time for such codes to be adopted and even longer for them to have an impact on patients [5, 11].

The general expectation among the public of how complaints are handled is not positive. More than half think that physicians will cover each other’s back and only a minority think that complaints are dealt with impartially through fair procedures. Patients who submit complaints will feel they have been wronged [2], which may exacerbate the sceptical outlook of general population about how their complaint will be handled. Nickerson [37] found that the natural tendency of people is to look for (and see) evidence that directly supports the expectations they hold. Research into service satisfaction based on the expectancy disconfirmation model makes clear that people tend to see what they think *will* happen, a tendency known as assimilation towards expectations [21, 37]. So even in situations where the procedures are fair and impartial and physicians do not cover for each other and where physicians *should* be open, patients are most likely to hear and experience what they think will happen. Oliver [21 (p. 112)] states that expectations will dominate a satisfaction decision when the actual performance is difficult to judge. A performance is difficult to judge when the objective performance is difficult to see, when performance is an ambiguous concept or when the procedures are complex. Procedures for complaints handling very often are complex and not always transparent. But, most important the performance of a complaints procedure is an ambiguous concept. For instance, when are apologies truly convincing? This is difficult to assess for a patient. Previously, Mazor et al. [35] found evidence for continuing differences between patients’ needs for apology and disclosure, and their experiences of actual apologies and disclosure conversations. They attribute this to the difficulty physicians have in expressing these apologies in a truly convincing way. Our study adds that patients may not notice these apologies, that are of themselves already difficult to make, because many of them do not expect that such apologies will be made.

Physicians express far more positive opinions about the way patients’ complaints are handled: most physicians share the opinion that complaints handling procedures are fair and impartial and that patients’ complaints are taken seriously. There are expectation gaps of around 50 % for these items. Such a gap means that when patient and physician meet up because of a complaint, they are likely to start off with opposite expectations. Many physicians will be unaware of the negative expectations of the complainant. Physicians, not unlike patient, will see what they expect to see: a fair procedure. Being unaware of these different expectations may lead to misunderstandings in both directions, in addition to the problems that were the reason for the complaint, irrespective of the good intentions of either party.

### Complaints and disclosure

True openness coming proactively from the physician might prevent complaints. A vital difference between complaints and open disclosure lies in the initiative. The initiative for disclosure comes from the care professional; the initiative for a complaint comes from the patient. However, studies that compare medical records and patients’ feedback about patient safety show that patients report other issues than those found in medical records [13, 15], suggesting that not all the issues patients might raise will be included by physicians in open disclosure. To pick up those issues that are relevant to patients, it remains essential that physicians actively enquire about patient safety and possible complaints. Most physicians in our study seem to agree with this view.

### Limitations and strengths of this study

This study is a cross-sectional study using questionnaires to elicit expectations of patients and physicians about complaints handling. This study does not assess the feelings and expectations of patients and physicians interacting with one another because of a complaint, nor does it assess the actual impact of sceptical expectations on the final satisfaction with complaints handling. However, the strength of this study lies in the insights it provides in the expectations of patients and physicians, irrespective of their present or past involvement with complaints handling. It provides insights about basic attitudes and shows that they are different and rather striking and therefore need to be taken seriously. A serious limitation is that the under-30 age group in the public is virtually absent from this study. The results of this study therefore do not reveal the expectations of this age group. For two items, we found that older people have more positive expectations about the way complaints are handled. This suggests that younger people might have more critical expectations of how complaints are handled. Another limitation is that this study covers only one country, the Netherlands. Nonetheless, this does not mean that the results of the study are relevant for the Netherlands only. Earlier studies in the Netherlands looking at complaints handling demonstrated results that are in line with international studies [4, 31]. The debate and the developments in patient safety and disclosure in the Netherlands are closely linked to the same debate in other countries (e.g. [38, 39]) as well as the existence of a code of conduct for complaints handling and openness. We therefore believe that the findings of our study are also relevant for many other countries that are investing in complaints handling and working on processes of disclosure. Finally, this study, as any survey, may have been subject to potential response bias and selection bias.

## Conclusions

This study demonstrates the existence of large expectation gaps between the public and physicians, with the majority of the public expressing negative expectations. We think this finding could be an additional explanatory factor for patients’ negative evaluations of complaints procedures, as patients are more likely to hear and experience what they expect will happen.

The relatively more negative expectations among the public will make it difficult to turn the actual experience of filing a complaint in a more positive way. This is also demonstrated by the fact that patients that did file a complaint less often said that complaints are dealt with through fair procedures or that complaints are taken seriously.

Mazor et al. [35] state that patients expect action, not only words. They may be right in suggesting that words alone will not overcome these negative expectations and that genuine actions are required. Healthcare complaints procedures could be marketed as being accessible, impartial and fair, to favourable influence expectation, followed by clear and indisputable evidence that this is indeed the case. This might lead to a situation where apologies, if they are made, are actually heard. Thus, possibly leading to a more positive evaluation of the experience of filing a complaint among patients.

## Additional file

10.1186/s13104-015-1479-z Questionnaire: expectations of complaints handling in health care.
