# Supplementary material for: Complaint handling in healthcare: expectation gaps between physicians and the public; results of a survey study
Source: BMC Res Notes. 2015 Oct 1;8:529. doi: 10.1186/s13104-015-1479-z (PMC4591727; doi:10.1186/s13104-015-1479-z)
Supplement: Supplementary file 1 — 10.1186/s13104-015-1479-z Questionnaire: expectations of complaints handling in health care. [file 13104_2015_1479_MOESM1_ESM.doc]

Questionnaire on complaints handling in health care (a translated version and the original version in Dutch)

Used in: Complaint handling in healthcare: expectation gaps between physicians and the public; results of a survey study. R D. Friele, P.M. Reitsma andJ.D. de Jong

English version (translated)

| **Health care complaints** |
| --- |

32. (patient questionnaire)

Did you even file a health care complaint? (for example about a physician or a hospital)?

no

yes

32. (physician questionnaire)

Did you ever receive a complaint from a patient about the delivery of care?

no

yes

33. To what degree do you agree with the following statements?

|  | *agree entirely* | *agree* | *agree nor disagree* | *disagree* | *entirely disagree* | *don’t know* |
| --- | --- | --- | --- | --- | --- | --- |
| a. For patients it is clear where to go with a complaint | ❑ | ❑ | ❑ | ❑ | ❑ | ❑ |
| b. Patient complaints are dealt with through fair procedures | ❑ | ❑ | ❑ | ❑ | ❑ | ❑ |
| c. Patient complaints are dealt with through fair procedures | ❑ | ❑ | ❑ | ❑ | ❑ | ❑ |
| d. Patient complaints are dealt with through fair procedures | ❑ | ❑ | ❑ | ❑ | ❑ | ❑ |
| e. In case of a patiënt's complaint, physicians cover each other's back | ❑ | ❑ | ❑ | ❑ | ❑ | ❑ |
| f. Physicians should ask, on their own initiative, whether things go well and about complaints | ❑ | ❑ | ❑ | ❑ | ❑ | ❑ |
| g. In health care, people are open when things went wrong with the treatment of patients | ❑ | ❑ | ❑ | ❑ | ❑ | ❑ |
|  |  |  |  |  |  |  |

Dutch version (original)

| **Klachten over de gezondheidszorg** |
| --- |

32. (patiënten vragenlijst)

Heeft u weleens een klacht ingediend over de gezondheidszorg (bijvoorbeeld over een arts of ziekenhuis)?

- nee
- ja

32. (artsen vragenlijst)

Heeft u wel eens klacht van een patiënt over de zorgverlening ontvangen?

- Nee
- ja

33. In hoeverre bent u het eens met de volgende stellingen?

|  | *geheel mee eens* | *mee*  *eens* | *eens noch oneens* | *oneens* | *geheel oneens* | *weet niet* |
| --- | --- | --- | --- | --- | --- | --- |
| a. Het is duidelijk waar patiënten met hun klachten terecht kunnen | ❑ | ❑ | ❑ | ❑ | ❑ | ❑ |
| b. Er zijn eerlijke procedures om klachten van patiënten te behandelen | ❑ | ❑ | ❑ | ❑ | ❑ | ❑ |
| c. Klachten van patiënten worden serieus genomen | ❑ | ❑ | ❑ | ❑ | ❑ | ❑ |
| d. Klachten van patiënten worden onpartijdig behandeld | ❑ | ❑ | ❑ | ❑ | ❑ | ❑ |
| e. Als een patiënt een klacht indient, houden artsen en ziekenhuizen elkaar de hand boven het hoofd | ❑ | ❑ | ❑ | ❑ | ❑ | ❑ |
| f. Artsen moeten uit zichzelf vragen of alles naar wens verloopt of dat er klachten zijn | ❑ | ❑ | ❑ | ❑ | ❑ | ❑ |
| g. In de gezondheidszorg is men er open over, wanneer iets niet goed is gegaan in de behandeling | ❑ | ❑ | ❑ | ❑ | ❑ | ❑ |
